# Amyloid Fibrils and Their Applications: Current Status and Latest Developments

**DOI:** 10.3390/nano15040255

**Published:** 2025-02-07

**Authors:** Bingxu Liu, Hongnan Zhang, Xiaohong Qin

**Affiliations:** Key Laboratory of Textile Science & Technology, College of Textiles, Donghua University, Ministry of Education, Shanghai 200051, China; 1229717@mail.dhu.edu.cn (B.L.); xhqin@dhu.edu.cn (X.Q.)

**Keywords:** nanomaterial, protein, amyloid, functionalization, fibril, nanofiber

## Abstract

Amyloid fibrils are one of the important forms of protein aggregates, first discovered in the pathological brain tissues of patients with various neurodegenerative diseases. They are considered the core pathological markers of different neurodegenerative diseases. In recent years, research has found that multiple proteins or peptides dynamically assemble to form functional amyloid-like nanofibrils under physiological conditions, exhibiting excellent mechanical properties, high environmental stability, and self-healing ability. Therefore, they have become a class of functional biological nanomaterials with important development potential. This article systematically reviews the latest progress in the preparation, functionalization, and application of amyloid-like nanofibrils in engineering and provides an outlook on possible future development directions.

## 1. Amyloid Proteins

Amyloid proteins were initially known in the study of certain diseases, which are closely related to protein misfolding, including systemic amyloidosis and a wide variety of neurodegenerative diseases, the most typical of which are Alzheimer’s and Parkinson’s diseases [1,2]. Amyloid proteins are rich in parallel or antiparallel arranged β-sheets composed of β-strands, which are then assembled into intersecting beta structures. After undergoing elongation to become fibrils, they are further assembled into amyloid fibrils. Due to the existence of β-sheets and widespread hydrogen bonding, the structure of amyloid proteins is very stable. In addition, due to differences in amino acid sequences, domain combinations, and environmental factors, amyloid proteins exhibit structural diversity. Currently, increasing evidence suggests that small soluble oligomers are the main cause of abnormal protein aggregation with cytotoxicity [3]. The pathogenic mechanism of amyloid protein varies in different diseases, mainly due to the following pathogenic factors: inhibiting protein degradation, breaking protein homeostasis, inducing dysfunction of important organelles such as mitochondria, damaging cell membranes, and self-replication and intercellular transmission of pathological fiber aggregation. Of course, not all amyloid proteins are related to diseases, such as human premelanosome amyloid proteins, milk proteins, etc. [4,5].

In some plants, there are also abundant amyloid proteins. Wheat gluten is rich in glutamine and hydrophobic amino acids and has a high β-folding tendency with an α-helix. These characteristics make it easy for wheat gluten to form amyloid fibrils [6]. In addition, plant seeds such as hemp seeds, rice, and soybeans are also rich in easily fibrillated amyloid proteins. Amyloid-like fibrils from plant-based food proteins have great potential for development in food and other biomaterials due to their functional properties. However, their low solubility and complex structure hinder their fibrosis and application [7,8,9,10].

Bacteria also produce amyloid nanofibrils to survive in harsh natural environments. For bacteria, amyloid nanofibrils are mainly present in biofilms and are a fundamental component to enhance the mechanical strength of the membrane structure, resist chemical and biological degradation, and ensure its structural integrity. For example, in Bacillus subtilis, the extracellular matrix (ECM) is mainly composed of functional amyloid protein TasA, which is both a part of the cell and helps regulate cell membrane dynamics, playing a crucial role in maintaining cell viability within the colony [11]. In Salmonella and Escherichia coli, amyloid fibrils mainly exist in the form of bacterial outer membrane porins, which have been shown to be associated with bacterial virulence. In addition, they mainly play a structural role, which is the same as in *Bacillus subtilis* [12,13]. The same theory applies to the biofilm of Pseudomonas and Staphylococcus aureus [14].

Under the premise of pursuing sustainable development, extracting amyloid protein to develop new types of functional materials is a very feasible choice. As a protein widely present in living organisms, amyloid protein can be artificially induced or influenced by environmental factors to form amyloid-like nanofibrils through processes such as folding, assembly, and aggregation (Table 1). Non-pathogenic amyloid nanofibrils have high transparency, excellent mechanical properties, and a surface rich in various amino acid residues, making them have a double-layer structure and amphiphilicity. In addition, they also have good biocompatibility. Therefore, they can be used as basic structural and functional units between themselves and other materials through electrostatic forces, hydrogen bonds, and π–π interactions to construct functional materials with different dimensions for applications in fields such as electrochemistry, biomedicine, biosensing, and photovoltaic cells.

## 2. Amyloid Fibrils

**Figure 1:**
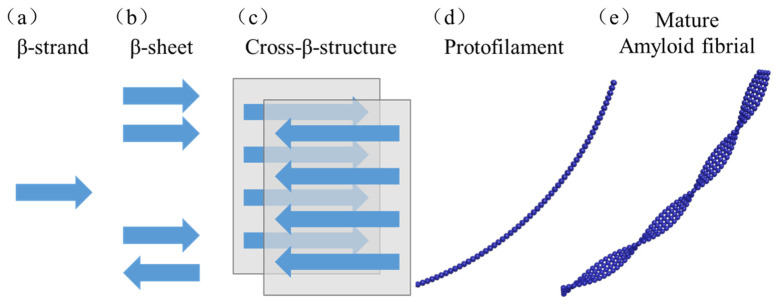
(**a**) β-strands in protein structure; (**b**) β-sheet structure; (**c**) cross-β-sheet structure composed of parallel or antiparallel arranged β-strands; (**d**) protofilaments formed by crossed beta structures; (**e**) the final formed amyloid fibrils.

Although proteins or peptides have highly stable macromolecular structures, factors such as pH, ionic strength, and temperature can still cause protein denaturation. Amyloid-like protein nanofibers are assembled and aggregated from denatured proteins or peptides, with a diameter of less than 100 nm and a highly ordered linear structure without branching. The numerous intersecting β-sheets and three-dimensional zipper structures in their microstructure endow them with stable performance in certain extreme environments (Figure 1) [20,21]. There are mainly two types: one type is pathological amyloid nanofibrils produced by organisms due to aging or environmental factors; the other type is nanofiber materials designed and modified by humans, with a significantly increased aspect ratio, which can be used to composite with other materials to prepare new functional materials.

### 2.1. Existence in Nature

The process of generating amyloid nanofibrils is dynamic and very complex. It is generally believed that proteins present in organisms undergo changes in their spatial conformation due to aging or environmental factors, leading to the appearance of β-sheets and ultimately forming amyloid nanofibrils. Most amyloid nanofibrils have a very stable structure because their spatial configuration is very similar to a zipper (Figure 1c). Amyloid fibrils exhibit extremely high similarity in molecular structure, which mainly depends on the inherent structure of the protein. Almost all proteins contain at least one self-complementary sequence that can form amyloid fibrils, which has been proven through extensive genomic investigations [22]. Different fibrotic conditions, such as temperature, pH, salt ions, and protein concentration, can lead to the emergence of different amyloid fibrillar variants. With the advancement of technology and equipment, researchers have begun to explore the formation mechanism of amyloid proteins in more depth. However, up to now, it is still in the stage of structural characterization of amyloid proteins, and there is still a lack of convincing conclusions about their specific formation mechanism.

### 2.2. Artificial Synthesis

**Figure 2:**
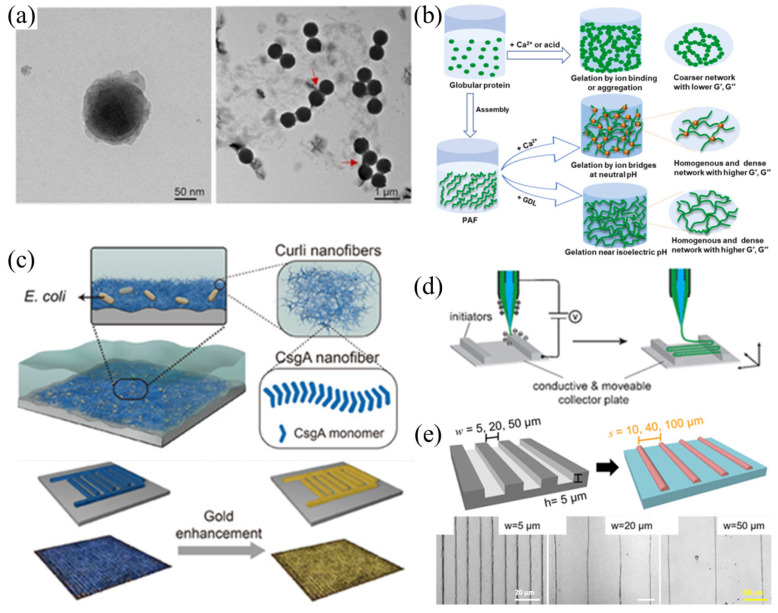
(**a**) Induction of amyloid-like fibrils synthesis by nanoparticles. The red arrow points to the place where amyloid fibers first form, reproduced from ref. [23], Elsevier. (**b**) Induction of amyloid-like fibrils synthesis by inorganic ions, reproduced from ref. [26], Elsevier. (**c**) Controllable preparation of amyloid fiber by programming, reproduced from ref. [29], Nature Publishing Group. (**d**) Development of amyloid fiber nanomaterials by electrospinning, reproduced from ref. [35], American Chemistry Society. (**e**) Preparation of amyloid fiber by spatial confinement of amyloid fiber, reproduced from ref. [42], American Chemistry Society.

After studying the formation process and characteristics of amyloid nanofibrils in living organisms, amyloid-like nanofibrils were obtained through artificial design and synthesis methods, including spinning, induced self-assembly, and other top-down or bottom-up methods. These nanofibers were then composited and assembled into functional materials of different dimensions and applied in various fields (Figure 2).

#### 2.2.1. Induced Synthesis

Some tiny particles have a certain impact on the aggregation of amyloid proteins and the formation of amyloid-like nanofibers. Polystyrene, as the most common nanoplastics, is harmful to human health. Studies have found that it plays a certain role in regulating the formation of amyloid-like fibrils and significantly promotes the primary nucleation step of amyloid-like fibrils [23,24]. Cellulose nanocrystals can regulate the gelation behavior of soy protein isolate amyloid-like fibers, and the binding between the two is mainly driven by non-covalent interactions. Their addition reduces the particle size, turbidity, subunit fragments, and crystallinity of soy protein isolate amyloid-like fibers, promotes the transition from alpha helix to β-sheet, improves thermal stability, exposes more amino acid residues, and increases intermolecular interactions [9].

Inorganic ions can also regulate the formation process of amyloid-like nanofibers. Biofilm-associated proteins are important surface proteins mainly involved in intercellular adhesion and mediating biofilm formation. Research results have shown that Staphylococcus Baps can regulate biofilm formation through sensing changes in pH and [Ca^2+^] concentration and amyloid-dependent regulation. However, its specific mechanism is still unknown [25]. Amyloid-like nanofibers derived from inexpensive food proteins and nanocellulose are renewable and biodegradable materials with wide applications in water purification and biomaterials [26]. Plant protein self-assembly into amyloid-like fibrils is a modification introduced in emerging food and material applications. The globulin portion of amaranth seeds is recovered from food waste through double salt extraction, and self-assembled into amyloid-like fibrils under acidic conditions through thermal induction. The amaranth plant protein waste is introduced into the raw material column of amyloid-like nanofibers [19]. Under strong acidic conditions, the effect of different salt ion concentrations on rice protein fibrillation was studied, providing new insights for the application of rice protein fibrillation in food [8,27].

#### 2.2.2. Programmable Controllable Backup

Li et al. prepared a modifiable thin film material to protect the coating substrate by utilizing gene-programmable CsgA amyloid proteins to self-assemble nanofibers into amyloid nanofibers in an aqueous solution [28]. It is a breakthrough to use genetic engineering and bioengineering to produce emerging biomaterials, but its 3D structure control has always been a difficulty. Through programming and self-assembly, amyloid monomers produced by genetically engineered cells can form nanofibers, which can further develop into networks and extrudable hydrogels. This approach has shown great promise for the 3D printing of living architectures [29]. The aggregation and deposition of proteins into amyloid fibers can induce cellular oxygen toxicity/iron toxicity, autophagy damage, mitochondrial dysfunction, production of reactive oxygen species, and membrane disruption. Therefore, the aggregation of normal proteins in cells may also have good anti-tumor effects. Peng et al. designed an EISA peptide that can self-assemble into amyloid aggregates under tyrosinase catalysis to inhibit the growth of tumor cells [30].

#### 2.2.3. Electrostatic Spinning

Amyloid-like fibers have generated steadily increasing traction in the development of natural and artificial materials. However, due to their inherent brittleness, directly constructing large amyloid-like protein membranes from amyloid-like protein fibers remains a challenge. Electrospinning technology is a good choice for preparing membrane materials due to its simple equipment, low cost, and controllable process [31,32]. A simple and universal method (phytic-acid aerosol spray process) for manufacturing macroscopic and adjustable amyloid-like protein films through rapid electrostatic self-assembly at the air–water interface was utilized, benefiting from the combination of amyloid fibrils and nanoparticles (such as conductive carbon nanotubes, magnetic iron oxide particles, etc.) to construct multifunctional amyloid-like protein films with adjustable properties, providing ideas for the manufacturing of related intelligent devices [33,34]. Assemble amyloid-like fibers using core–shell electrospinning with micro constraints (low voltage core–shell electrospinning). The composition of the fiber shell layer is polymer, and the core layer is composed of high-density aggregated amyloid-like fibrils [35]. Accurately manufacturing artificially designed molecular complexes into ordered structures similar to natural counterparts will have wide applications and is currently a major challenge. Amyloid-like proteins containing chitin-binding domains were prepared through genetic engineering. By combining their bottom-up self-assembly with top-down manufacturing methods such as electrospinning, a series of unique independent structures with rich and diverse functions were generated, such as light conduction and the ability to mimic extracellular matrix. With the participation of genetic engineering, their functions can be adjusted, and their application prospects are even broader [36].

#### 2.2.4. Others

Due to its molecular function, amyloid protein is currently being regarded as a very promising nanotechnology building block. The self-assembly of amyloid protein is closely related to its arrangement and orientation. Currently, the concentration of amyloid protein water dispersion can be adjusted using its liquid crystal properties [37,38]. There are currently two main control methods for it: one is to solve the problem of the directional vector field by making use of steady extensional flows [39]; the other is geometric constraint, which can accurately control the equilibrium configuration of the original fiber cholesterol phase [40,41]. In addition, controlling the evaporation rate of the water dispersion can simultaneously achieve the solidification of amyloid-like fibers and anisotropic aggregation. When the liquid crystal phase of the fiber structure is limited by the geometric constraints of the model, its arrangement is greatly improved. The obtained ordered aggregates are used to form an array of gold nanoparticles, which exhibit strong conductivity [42].

## 3. Research Progress on Functionalization of Amyloid-like Nanofibers

**Figure 3:**
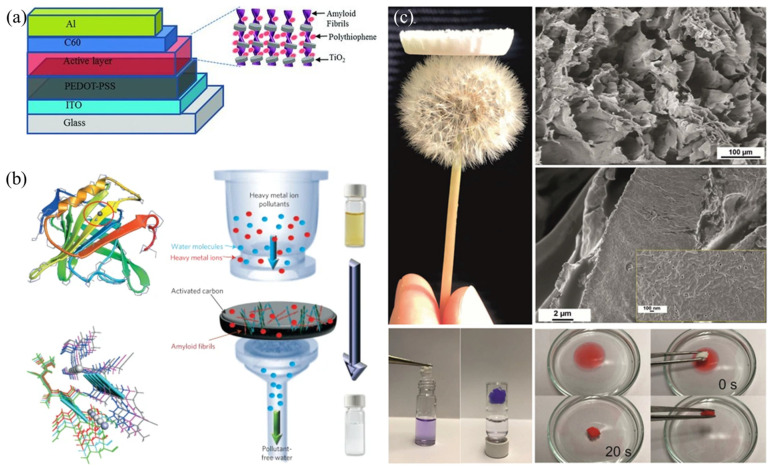
(**a**) Modification of amyloid fibrils, reproduced from ref. [43], Wiley. (**b**) Processing of amyloid fibrils into two-dimensional membrane materials, reproduced from ref. [49], Nature Publishing Group. (**c**) Development of three-dimensional gel materials using amyloid fibrils, reproduced from ref. [52], Wiley.

Due to the fact that amyloid proteins can be artificially synthesized, it is possible to purposefully utilize aggregates, making bottom-up assembly based on structural doping and functionalization possible (Figure 3) [35].

### 3.1. One-Dimensional

The modification of materials with functional nanoparticles is a commonly used functionalization method. Using protein fibers as templates, densely packed titanium dioxide nanoparticles are generated on the surface of amyloid fibrils through precursor reactions, forming amyloid protein hybrid nanowires. These nanowires are then complexed with water-soluble semiconductor thiophene to act as electron donors and acceptors, respectively. They can be used in heterojunction photovoltaic devices and, in the future, in organic electronics and hybrid solar cells [43,44]. Grafting functional units onto the target in the form of grafting is also one of the commonly used methods for functionalization. Polydopamine is used to graft polyethyleneimine(PEI) onto amyloid fibrils to form a hybrid membrane, which is used to treat radioactive isotopes in water. The resulting material shows a good preference for TcO_4_^−^ and a good adsorption effect under acidic and alkaline conditions and has good application prospects in water treatment for hospitals and nuclear power plants [45,46]. It is also a good functional choice to achieve modification by simple treatment and hydrogen bonding force. Modification of maltodextrin on the surface of β-lactoglobulin amyloid-like nanofibers can improve the utilization rate in subsequent preparation, such as lotion or dispersion [47,48].

### 3.2. Two-Dimensional

Filtering or precipitation is a very simple method for preparing composite materials without considering the interactions between components. By vacuum filtering, the solution of amyloid fibrils was mixed with activated carbon. The stiffness and viscosity of amyloid fibrils make it easy to form a composite membrane. In addition, the presence of amyloid fibrils allows the fixed heavy metal ions to be reduced by high temperature or chemical means [49]. By using the anti-solvent precipitation method and adjusting the pH of the synthesis process, a fruit tree-shaped composite structure was prepared with lysozyme amyloid protein as the line and zein aggregates as the beads. This composite material is more stable than generally shaped composite materials and provides constructive information for the manufacture of amyloid fibrils/soluble protein composite materials [50]. As mentioned earlier, some materials have a certain inducing effect on the aggregation of amyloid proteins. Taking advantage of this, unexpected results can be achieved in the development of new functional materials. Due to its ability to generate static electricity, reduce aggregation, and facilitate the fixation of biomolecules, graphene acetylene material has been applied in various biological applications. γ-graphyne, which can be artificially designed with a natural bandgap, was used to prepare few-layer γ-graphyne as an electrostatic semiconductor through a two-step method. Its electrostatic properties induce the growth of amyloid-like fibers with high aspect ratios, ensuring the survival rate of cells on the surface and neuronal differentiation [51].

### 3.3. Three-Dimensional

Low dimensional materials have considerable advantages in the field of functional materials due to size effects, but in practical applications, the transition from micro to macro is an insurmountable problem. It is of great significance to develop amyloid protein or amyloid fibrils into three-dimensional materials. At present, certain breakthroughs have been made in the development of gel materials [52,53]. Multifunctional nanofiber materials were prepared by combining the widely existing whey protein with chitin, and different gel materials were obtained through design and assembly, which were used as paramagnetic materials and catalyst carriers [54,55]. Electrostatic interaction plays a very important role in the combination of composite materials. In the gel material of amyloid protein and nanocellulose, the crosslinking of the two enhances the elasticity of the formed network and improves the mechanical properties of functional hybrid materials based on these two biopolymers [56]. Due to its mechanical robustness, biocompatibility, and nanometer size, amyloid fibril has been considered a potential nanomaterial for biomedical applications. By forming a fixed disulfide bond on the surface of amyloid fibril formed by α-synuclein protein and introducing cysteine residues into its C-terminus, it can fully extend, prevent breakage due to thermal waves, and improve its mechanical properties. Using this fibril, self-healing hydrogels and aerogels for dye absorption can be prepared [57]. The amyloid fibril from protein waste can be used as a functional scaffold for biomimetic mineralization of metal–organic frameworks, and the resulting amyloid fibril/ZIF-8 mixed air gel can remove nine different heavy metal ions from water due to its layered structure [58].

## 4. Application Based on Amyloid Nanofibrils

Amyloid nanofibrils have many excellent properties and are easy to functionalize. They have many potential application areas, including but not limited to pharmaceuticals, the food industry, cosmetics, and environmental protection. Overall, the application prospects are very broad and have important economic and social significance.

### 4.1. Water Treatment

The purpose of water treatment is to improve water quality to meet certain standards, which is of great significance for the development of industrial production, the improvement of product quality, and the maintenance of ecological balance [59,60]. Directly using protein fibrils as coagulants can treat water pollution. Lysozyme amyloid protein, as a new type of natural biological flocculant, has a positive surface charge over a very wide pH range, making it effective in removing polystyrene, humic acid, and large particulate matter [61,62]. A unique vine-shaped three-dimensional porous fiber membrane was prepared by integrating carbon nanotubes into bovine serum albumin amyloid-like fibrils. Specifically, carbon nanotubes were embedded as the basic framework, and the unique network microstructure can minimize water resistance and improve the availability of active sites. The results showed that the composite film has adsorption capacity, rapid kinetics, and reusability for Congo red far beyond the general level [63,64]. The ZrO_2_ hybrid membrane of amyloid fibrils and carbon can be used for the treatment of fluorine-containing wastewater, and its performance far exceeds that of commercial aluminum membranes. Moreover, after saturated membrane regeneration, its performance will not decrease due to regeneration [65,66].

### 4.2. Biomedicine

The secretory granules of the mammalian endocrine system are functional amyloid proteins, which can be used as dynamic libraries to store protein hormones and release them into the bloodstream. If amyloid nanofibers are designed to mimic the function of secretory granules to achieve functional storage and release of nanoparticles, they will have a very broad prospect in clinical and industrial applications [67,68]. The oligomers formed by amyloid protein in the early stages of aggregation have certain cytotoxicity, and accelerating the aggregation process can reduce the total toxicity of oligomers to cells. Experiments have shown that using eight-residue peptides can accelerate the aggregation of unrelated proteins into fibrils, thereby reducing cytotoxicity. This is considered a promising strategy [69].

### 4.3. Biocatalyst

Enzymes, as essential substances in living organisms, play an important role in numerous biochemical reactions, enabling them to proceed efficiently even under mild conditions. Amyloid-like protein nanofibers also have enzymatic catalytic functions in some cases [70,71]. Glucagon is a key peptide hormone that helps control blood glucose levels and lipid metabolism. Although the formation of glucagon amyloid fibrils has been documented, its biological function remains a mystery. Experimental evidence suggests that glucagon amyloid fibrils can act as catalysts in various biological reactions, including ester hydrolysis, lipid hydrolysis, and dephosphorylation [72]. Enzymes typically fold into clear 3D protein structures, exhibiting high catalytic efficiency and selectivity. It has been suggested that the earliest enzymes may have been generated by short peptides self-assembling into supramolecular amyloid-like structures [73]. Several artificial amyloid proteins have been proven to have catalytic activity and advantages over natural enzymes in modularity, flexibility, stability, and reusability. Recently, four different polar prion-inspired peptide self-assembled amyloid-like fibrils have been reported, which can serve as both metal cleaners and nanoenzymes, fully demonstrating the multifunctionality of prion-inspired peptide assemblies [74].

### 4.4. Structural Control

Amyloid-like fibers can control the structure of materials at both macroscopic and microscopic levels to meet different needs [75]. Artificial soy protein meat and animal stem cell-based artificial meat have great application prospects for alleviating food crises, as well as environmental and animal protection issues. In this process, sustainable and edible scaffolds are a major breakthrough in solving the problem of artificial meat production. The three-dimensional porous scaffold of artificial meat is produced by crosslinking soy protein amyloid-like fibers prepared by microbial transglutaminase and temperature-controlled steam annealing technology and has been proven to successfully promote the proliferation and differentiation of mouse skeletal muscle myoblasts [10,76]. Microscopically, nanofibrils can improve the cohesion of the assembly of superparticles, but the morphology of the customized superparticles added to nanofibrils has not been well developed at present. β-lactoglobulin amyloid-like fibrils have been studied for the shape control of the assembly process of spray-dried superparticles composed of silica nanoparticles. As a result, the transformation of superparticle morphology from circular to corrugated has been achieved, which can be used as an effective additive for spray-dried superparticle morphology engineering, and is important for many applications [77,78].

### 4.5. Pressure Sensors

Pressure sensors have been applied to various fields from biomedicine to aerospace engineering. However, it is still difficult to develop pressure sensors with high mechanical strength, high accuracy, and high stability at the same time. By combining amyloid fibril aerogels made of α-synuclein self-assembled protein with multi-wall carbon, a highly robust and sensitive pressure sensor has been developed. Aerogels provide a 3D network for the structure, and multi-wall carbon nanotubes are used as reinforcement materials to improve the mechanical and electrical properties of the device. It exhibits stable sensing ability for human movement, airflow, and underwater pressure and can be used for the development of wearable devices and artificial skin [79,80,81].

### 4.6. Others

It can be used as a bioplastic. By extracting protein with alkali from rapeseed cake containing cruciferous elements and using self-assembly, mature amyloid-like protein fibers are obtained. Then, polyvinyl alcohol and glycerol are added to obtain environmentally friendly bioplastics, which have low water absorption and good mechanical properties, meeting the urgent need to replace plastic products with renewable materials to reduce environmental burden [82,83]. It can also be used as a lotion stabilizer. Amyloid protein has been found to have ice recrystallization inhibitory activity, but the activity is relatively moderate and needs to be improved. Research shows that when amyloid protein is used as a stabilizer for Pickering emulsion, the Pickering emulsion droplets are formed at the oil–water interface, and its activity is greatly enhanced and is proportional to the particle size and the number of solution drops. This discovery is of great significance for improving the quality of frozen food [84,85].

## 5. Conclusions and Prospects

In recent years, with the improvement of technology and the popularization of green and environmental protection concepts, the functional treatment technology of starch-like fibers has been continuously innovated. The application fields are constantly expanding, covering multiple areas such as textiles, food, medicine, and environmental protection. Starch-like fibers are widely used in the manufacturing of biodegradable textiles in the textile industry; in the food industry, they are used for producing food packaging materials and additives; in the field of medicine, they are used to manufacture drug sustained-release agents and tissue engineering scaffolds; in the field of environmental protection, they play an important role in the manufacturing of water treatment materials and adsorbents. Overall, the functionalization progress and application status of amyloid fibers are in a flourishing stage, bringing innovation to traditional industries and injecting new vitality into the environmental protection industry, with broad market prospects and development potential.

However, from a yield perspective, the content of amyloid protein in animals and plants is much higher than that in bacteria. Therefore, extracting amyloid protein or amyloid fibrils from food waste or agricultural products will be the main source of raw materials in the future. However, the role of amyloid protein in plants is still unclear, and the extraction conditions are relatively strict. Therefore, researchers need to continue their efforts. The future direction of functionalization and application will mainly focus on the following aspects:

Materials Science: Research on the mechanical properties and thermal stability of amyloid fibers, develop high-performance materials for industry and construction, and utilize the self-assembly characteristics of amyloid fibers to design new nanomaterials and metamaterials for applications in fields such as optoelectronics and catalysis.

Environmental Science: Exploring the application of amyloid fibers in water treatment and air purification, utilizing their adsorption properties to remove heavy metal ions and organic pollutants.

Food Science: Research on the application of amyloid fibers in food as a thickener, stabilizer, or nutrient to enhance the functionality and taste of food.

Biotechnology: Using genetic engineering techniques to design and synthesize amyloid-like fibers with specific functions to meet specific biotechnology needs.

Biomedical applications: Utilizing the biocompatibility and adjustability of amyloid fibers to develop novel drug carriers to improve drug targeting and release efficiency. Using amyloid fibers for the development of biosensors, utilizing their specific binding ability to detect biomarkers or pathogens. These research directions will promote the application of amyloid fibers in various fields and advance the development of related technologies.

## Figures and Tables

**Table 1:** Sources of amyloid proteins in nature.

Source of Amyloid Proteins		Function
Bacteria	*Bacillus subtilis* [11]	Improving the mechanical strength of biofilm and resisting chemical and biological degradation
	*Salmonella* [12]	
	*Escherichia coli* [13]	
	*Pseudomonas aeruginosa* [14]	
	*Staphylococcus aureus* [14]	
Animal	*Premelanosome protein* [15]	Forming amyloid fibrils and promoting melanin deposition
	Parkinson’s disease [16,17]	Accumulation in the brain, triggering molecular cascades
	Alzheimer’s disease [16,17]	
	*Acorn barnacles* [18]	Regulating biomineralization and promoting the formation of fibrils
Plant	*Wheat gluten* [6]	Unclear till now
	Hemp seed [7]	
	*Oryza sativa* [8]	
	*Soybean* [10]	
	Amaranth seeds [19]	

## Abbreviations

The following abbreviations are used in this manuscript:

ECM	extracellular matrix
EISA	enzyme-instructed self-assembly
ZIF	zeolitic imidazolate frameworks
PEI	polyethyleneimine
